# When to Operate in Appendicitis

**Published:** 1903-01-24

**Authors:** 


### WHEN TO OPERATE IN APPENDICITIS.

Summing up the considerations which determine the necessity or otherwise for operation in appendicitis, Mr. Gilbert Barling[1] says that one should be guided always by the severity of those symptoms which are present, and should not be influenced by the absence of symptoms which are usually met with. If a patient does not vomit, and if his temperature is approximately normal, while the pulse is 120 and the abdomen very rigid, lay stress on these last two signs and pay little heed to the others; or again, if the pulse is but moderately quick, and the temperature is in correspondence, yet the patient continues to vomit despite the prohibition of mouth feeding, too great emphasis can hardly be placed on the last symptoms. If the temperature is falling and the pulse at the same time quickening, whatever the state of the other symptoms, the pulse will be the index to the condition inside the patient's abdomen.

[1] Brit. Med. Jour., Jan. 10.

